# Bis(μ-4-hydroxy­benzoato-κ^2^ *O*:*O*′)bis­[triaqua­bis(4-hydroxy­benzoato)-κ*O*;κ^2^ *O*,*O*′-terbium(III)] deca­hydrate

**DOI:** 10.1107/S1600536809052489

**Published:** 2009-12-12

**Authors:** Yi-Min Zhu, Pei-Pei Feng, Yang-Yi Yang, Seik Weng Ng

**Affiliations:** a MOE Key Laboratory of Bioinorganic and Synthetic Chemistry, School of Chemistry & Chemical Engineering, Sun Yat-Sen University, Guangzhou 510275, People’s Republic of China; b Department of Chemistry, University of Malaya, 50603 Kuala Lumpur, Malaysia

## Abstract

The title dinuclear compound, [Tb_2_(C_7_H_5_O_3_)_6_(H_2_O)_6_]·10H_2_O, lies on a center of inversion and the two Tb^III^ atoms are bridged by two 4-hydroxy­benzoate anions; each metal atom is further coordinated by one monodentate anion and chelated by the third anion. The eight-coordinate geometry approximates a square anti­prism. Hydrogen bonds of the O—H⋯O type connect the uncoordinated water mol­ecules to the dinuclear species, forming a three-dimensional network.

## Related literature

For a related structure, Tb_2_(H_2_O)_2_(DMF)_2_(C_7_H_5_O_3_)_6_, see: Zhou *et al.* (2008 ▶).
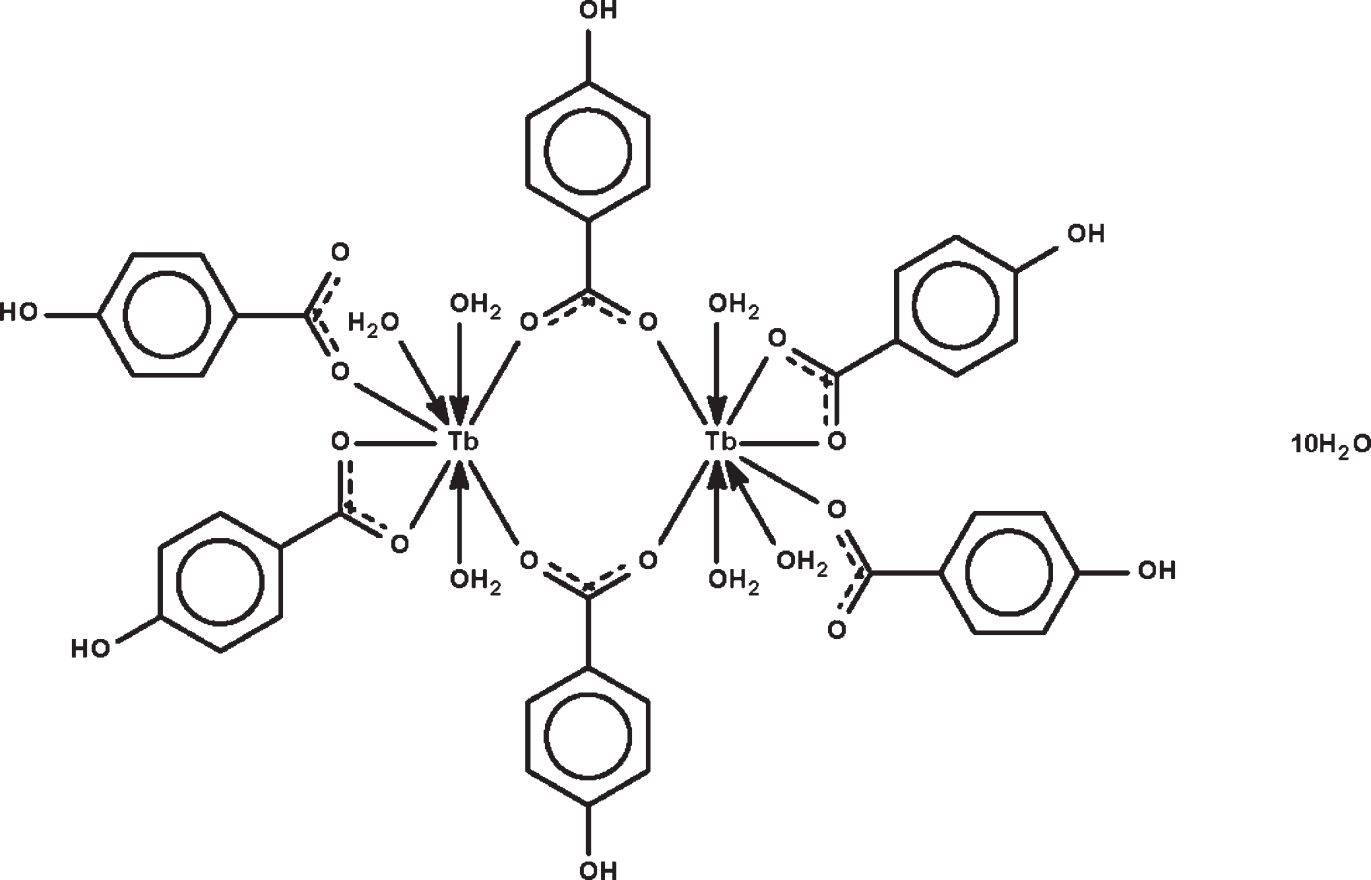


## Experimental

### 

#### Crystal data

[Tb_2_(C_7_H_5_O_3_)_6_(H_2_O)_6_]·10H_2_O
*M* *_r_* = 1428.76
Triclinic, 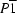

*a* = 10.8308 (5) Å
*b* = 11.3337 (6) Å
*c* = 11.5128 (6) Å
α = 90.463 (1)°
β = 101.690 (1)°
γ = 105.249 (1)°
*V* = 1332.37 (12) Å^3^
*Z* = 1
Mo *K*α radiation
μ = 2.73 mm^−1^
*T* = 173 K
0.47 × 0.30 × 0.19 mm

#### Data collection

Bruker SMART area-detector diffractometer
Absorption correction: multi-scan (*SADABS*; Sheldrick, 1996 ▶) *T*_min_ = 0.360, *T*_max_ = 0.625
12836 measured reflections
5756 independent reflections
5391 reflections with *I* > 2σ(*I*)
*R*_int_ = 0.018

#### Refinement

*R*[*F* ^2^ > 2σ(*F* ^2^)] = 0.018
*wR*(*F* ^2^) = 0.059
*S* = 1.09
5756 reflections
409 parameters
27 restraints
H atoms treated by a mixture of independent and constrained refinement
Δρ_max_ = 0.67 e Å^−3^
Δρ_min_ = −0.64 e Å^−3^

### 

Data collection: *SMART* (Bruker, 2001 ▶); cell refinement: *SAINT* (Bruker, 2001 ▶); data reduction: *SAINT*; program(s) used to solve structure: *SHELXS97* (Sheldrick, 2008 ▶); program(s) used to refine structure: *SHELXL97* (Sheldrick, 2008 ▶); molecular graphics: *X-SEED* (Barbour, 2001 ▶); software used to prepare material for publication: *publCIF* (Westrip, 2009 ▶).

## Supplementary Material

Crystal structure: contains datablocks I, global. DOI: 10.1107/S1600536809052489/xu2706sup1.cif

Structure factors: contains datablocks I. DOI: 10.1107/S1600536809052489/xu2706Isup2.hkl

Additional supplementary materials:  crystallographic information; 3D view; checkCIF report

## Figures and Tables

**Table 1:** Hydrogen-bond geometry (Å, °)

*D*—H⋯*A*	*D*—H	H⋯*A*	*D*⋯*A*	*D*—H⋯*A*
O3—H3⋯O7*w*^i^	0.84 (1)	1.82 (2)	2.612 (3)	158 (3)
O6—H6⋯O6*w*^ii^	0.83 (1)	1.87 (1)	2.678 (3)	165 (4)
O9—H9⋯O4*w*^iii^	0.84 (1)	1.98 (2)	2.763 (3)	156 (3)
O1*w*—H11⋯O4*w*	0.84 (1)	2.04 (1)	2.870 (3)	171 (3)
O1*w*—H12⋯O9^iv^	0.83 (1)	1.96 (1)	2.766 (3)	164 (3)
O2*w*—H22⋯O5*w*	0.83 (1)	1.86 (1)	2.678 (3)	167 (3)
O2*w*—H21⋯O5*w*^v^	0.84 (1)	2.20 (2)	2.952 (3)	150 (3)
O3*w*—H31⋯O3^ii^	0.84 (1)	1.95 (1)	2.777 (3)	171 (3)
O3*w*—H32⋯O7^vi^	0.84 (1)	2.25 (2)	2.916 (2)	137 (3)
O4*w*—H41⋯O8*w*^vii^	0.84 (1)	1.94 (1)	2.753 (3)	163 (3)
O4*w*—H42⋯O5^viii^	0.84 (1)	2.10 (1)	2.927 (3)	169 (3)
O5*w*—H52⋯O7*w*	0.85 (1)	2.01 (2)	2.811 (3)	157 (3)
O5*w*—H51⋯O8*w*^ix^	0.86 (1)	1.96 (1)	2.791 (3)	163 (3)
O6*w*—H61⋯O2	0.85 (1)	1.90 (1)	2.743 (2)	172 (3)
O6*w*—H62⋯O5	0.84 (1)	1.95 (1)	2.785 (2)	171 (3)
O7*w*—H71⋯O6*w*	0.84 (1)	1.90 (1)	2.732 (3)	172 (3)
O7*w*—H72⋯O5^x^	0.83 (1)	1.89 (1)	2.725 (3)	175 (3)
O8*w*—H81⋯O1^xi^	0.84 (1)	2.10 (2)	2.818 (3)	144 (3)
O8*w*—H82⋯O6	0.84 (1)	1.90 (1)	2.713 (3)	164 (3)

Symmetry codes: (i) 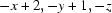; (ii) 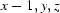; (iii) 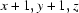; (iv) 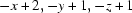; (v) 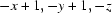; (vi) 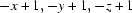; (vii) 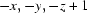; (viii) 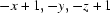; (ix) 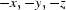; (x) 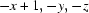; (xi) 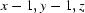.

**Fig. 1.**
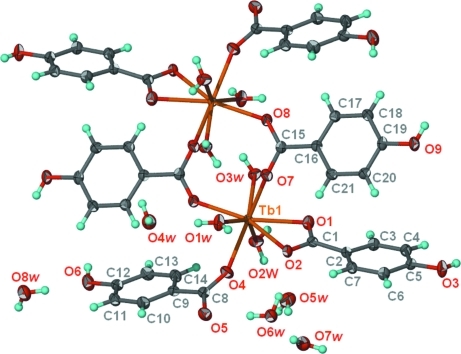
Thermal ellipsoid plot (Barbour, 2001) of Tb2(H2O)6(C7H5O3)6.10H2O at the 70% probability level; hydrogen atoms are drawn as sphere of arbitrary radius.

**Fig. 2.**
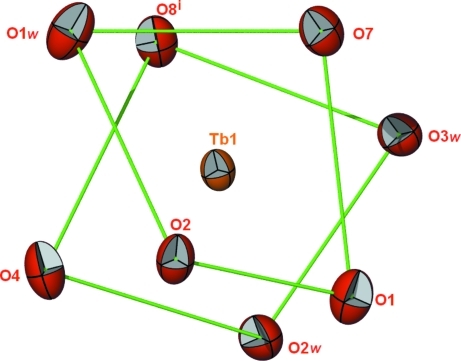
Detail of the coordination environment of the terbium(III) atom.

## supplementary crystallographic information

### Experimental

4-Hydroxybenzoic acid (0.3 mmol) and sodium hydroxide (0.3 mmol) were dissolved in water (5 ml) and this was mixed with a solution of terbium chloride (0.1 mmol) dissolved in water (5 ml). The white precipitate that formed was removed by filtration. Colorless crystals were isolated from the filtrated after two weeks.

### Refinement

Carbon-bound hydrogen atoms were generated geometrically and were constrained to ride on their parent atoms [C–H = 0.95, *U*_iso_(H) = 1.2*U*_eq_(C)]. The hydroxy and water H-atoms were located in a difference Fourier map, and were refined with distance restraints of O–H 0.84±0.01 Å and H···H 1.37±0.01 Å. Their temperature factors were tied by a factor of 1.5.

### Crystal data

**Table:**

[Tb_2_(C_7_H_5_O_3_)_6_(H_2_O)_6_]·10H_2_O	*Z* = 1
*M**_r_* = 1428.76	*F*(000) = 716
Triclinic, *P*1	*D*_x_ = 1.781 Mg m^−^^3^
Hall symbol: -P 1	Mo *K*α radiation, λ = 0.71073 Å
*a* = 10.8308 (5) Å	Cell parameters from 5392 reflections
*b* = 11.3337 (6) Å	θ = 2.3–27.0°
*c* = 11.5128 (6) Å	µ = 2.73 mm^−^^1^
α = 90.463 (1)°	*T* = 173 K
β = 101.690 (1)°	Block, colorless
γ = 105.249 (1)°	0.47 × 0.30 × 0.19 mm
*V* = 1332.37 (12) Å^3^	

### Data collection

**Table:**

Bruker SMART area-detector diffractometer	5756 independent reflections
Radiation source: fine-focus sealed tube	5391 reflections with *I* > 2σ(*I*)
graphite	*R*_int_ = 0.018
φ and ω scans	θ_max_ = 27.0°, θ_min_ = 1.8°
Absorption correction: multi-scan (*SADABS*; Sheldrick, 1996)	*h* = −13→13
*T*_min_ = 0.360, *T*_max_ = 0.625	*k* = −13→14
12836 measured reflections	*l* = −14→14

### Refinement

**Table:**

Refinement on *F*^2^	Primary atom site location: structure-invariant direct methods
Least-squares matrix: full	Secondary atom site location: difference Fourier map
*R*[*F*^2^ > 2σ(*F*^2^)] = 0.018	Hydrogen site location: inferred from neighbouring sites
*wR*(*F*^2^) = 0.059	H atoms treated by a mixture of independent and constrained refinement
*S* = 1.09	*w* = 1/[σ^2^(*F*_o_^2^) + (0.0407*P*)^2^ + 0.0925*P*] where *P* = (*F*_o_^2^ + 2*F*_c_^2^)/3
5756 reflections	(Δ/σ)_max_ = 0.001
409 parameters	Δρ_max_ = 0.67 e Å^−^^3^
27 restraints	Δρ_min_ = −0.64 e Å^−^^3^

### Fractional atomic coordinates and isotropic or equivalent isotropic displacement parameters (Å^2^)

**Table:**

	*x*	*y*	*z*	*U*_iso_*/*U*_eq_	
Tb1	0.521099 (9)	0.392335 (9)	0.316629 (8)	0.01393 (5)	
O1	0.68247 (16)	0.52500 (16)	0.22113 (15)	0.0219 (4)	
O2	0.71938 (16)	0.35118 (15)	0.28025 (15)	0.0189 (3)	
O3	1.25530 (17)	0.60503 (18)	0.13235 (17)	0.0243 (4)	
H3	1.262 (3)	0.6687 (19)	0.095 (3)	0.036*	
O4	0.44616 (17)	0.20553 (16)	0.21445 (16)	0.0233 (4)	
O5	0.44536 (17)	0.00991 (16)	0.21119 (16)	0.0236 (4)	
O6	−0.15974 (18)	−0.03603 (17)	0.16884 (18)	0.0280 (4)	
H6	−0.192 (3)	0.023 (2)	0.171 (3)	0.042*	
O7	0.66267 (16)	0.51402 (16)	0.47709 (14)	0.0207 (3)	
O8	0.65933 (16)	0.66801 (16)	0.59555 (15)	0.0209 (4)	
O9	1.23480 (17)	0.85877 (17)	0.50180 (16)	0.0220 (4)	
H9	1.262 (3)	0.9320 (13)	0.529 (3)	0.033*	
O1w	0.56576 (17)	0.25542 (17)	0.47360 (16)	0.0241 (4)	
H11	0.509 (2)	0.206 (2)	0.502 (3)	0.036*	
H12	0.6353 (16)	0.234 (3)	0.487 (3)	0.036*	
O2w	0.40382 (18)	0.41807 (17)	0.12181 (15)	0.0233 (4)	
H21	0.405 (3)	0.4895 (13)	0.102 (2)	0.035*	
H22	0.426 (3)	0.380 (2)	0.071 (2)	0.035*	
O3w	0.44922 (17)	0.57723 (16)	0.32029 (15)	0.0201 (3)	
H31	0.397 (2)	0.593 (3)	0.2625 (14)	0.030*	
H32	0.428 (3)	0.593 (3)	0.3838 (13)	0.030*	
O4w	0.39635 (19)	0.08545 (18)	0.59456 (16)	0.0267 (4)	
H41	0.358 (3)	0.130 (3)	0.624 (2)	0.040*	
H42	0.450 (3)	0.066 (3)	0.6490 (19)	0.040*	
O5w	0.4872 (2)	0.3305 (2)	−0.05591 (17)	0.0309 (4)	
H51	0.421 (2)	0.294 (3)	−0.111 (2)	0.046*	
H52	0.530 (3)	0.278 (2)	−0.037 (3)	0.046*	
O6w	0.69870 (17)	0.12258 (17)	0.18465 (16)	0.0235 (4)	
H61	0.711 (3)	0.1957 (12)	0.211 (3)	0.035*	
H62	0.6246 (17)	0.082 (2)	0.194 (3)	0.035*	
O7w	0.6606 (2)	0.18314 (17)	−0.04660 (16)	0.0286 (4)	
H71	0.676 (3)	0.160 (3)	0.0230 (11)	0.043*	
H72	0.627 (3)	0.1215 (18)	−0.0936 (19)	0.043*	
O8w	−0.30680 (19)	−0.22701 (18)	0.26414 (18)	0.0301 (4)	
H81	−0.280 (3)	−0.2897 (17)	0.276 (3)	0.045*	
H82	−0.249 (2)	−0.172 (2)	0.242 (3)	0.045*	
C1	0.7539 (2)	0.4523 (2)	0.2315 (2)	0.0169 (5)	
C2	0.8802 (2)	0.4855 (2)	0.19285 (19)	0.0163 (4)	
C3	0.9139 (2)	0.5908 (2)	0.1316 (2)	0.0190 (5)	
H3A	0.8513	0.6352	0.1057	0.023*	
C4	1.0383 (2)	0.6307 (2)	0.1084 (2)	0.0186 (5)	
H4	1.0609	0.7020	0.0660	0.022*	
C5	1.1296 (2)	0.5662 (2)	0.1474 (2)	0.0177 (5)	
C6	1.0956 (2)	0.4574 (2)	0.2042 (2)	0.0188 (5)	
H6A	1.1572	0.4112	0.2268	0.023*	
C7	0.9713 (2)	0.4181 (2)	0.22698 (19)	0.0178 (5)	
H7	0.9475	0.3446	0.2661	0.021*	
C8	0.3871 (2)	0.0935 (2)	0.2068 (2)	0.0184 (5)	
C9	0.2416 (2)	0.0589 (2)	0.1919 (2)	0.0168 (4)	
C10	0.1763 (2)	−0.0536 (2)	0.2258 (2)	0.0206 (5)	
H10	0.2237	−0.1109	0.2542	0.025*	
C11	0.0414 (2)	−0.0831 (2)	0.2184 (2)	0.0224 (5)	
H11A	−0.0027	−0.1592	0.2440	0.027*	
C12	−0.0276 (2)	−0.0015 (2)	0.1740 (2)	0.0201 (5)	
C13	0.0352 (2)	0.1103 (2)	0.1364 (2)	0.0214 (5)	
H13	−0.0135	0.1651	0.1034	0.026*	
C14	0.1704 (2)	0.1410 (2)	0.1477 (2)	0.0207 (5)	
H14	0.2146	0.2185	0.1251	0.025*	
C15	0.7152 (2)	0.6180 (2)	0.53115 (19)	0.0159 (4)	
C16	0.8513 (2)	0.6851 (2)	0.52099 (19)	0.0153 (4)	
C17	0.9058 (2)	0.8072 (2)	0.56448 (19)	0.0167 (4)	
H17	0.8550	0.8494	0.5982	0.020*	
C18	1.0337 (2)	0.8673 (2)	0.5587 (2)	0.0187 (5)	
H18	1.0704	0.9503	0.5883	0.022*	
C19	1.1080 (2)	0.8052 (2)	0.5093 (2)	0.0177 (5)	
C20	1.0543 (2)	0.6841 (2)	0.4635 (2)	0.0187 (5)	
H20	1.1045	0.6427	0.4282	0.022*	
C21	0.9260 (2)	0.6245 (2)	0.4702 (2)	0.0176 (5)	
H21A	0.8890	0.5418	0.4398	0.021*	

### Atomic displacement parameters (Å^2^)

**Table:**

	*U*^11^	*U*^22^	*U*^33^	*U*^12^	*U*^13^	*U*^23^
Tb1	0.01190 (7)	0.01180 (7)	0.01807 (7)	0.00220 (4)	0.00446 (4)	0.00005 (4)
O1	0.0188 (8)	0.0190 (9)	0.0318 (9)	0.0082 (7)	0.0104 (7)	0.0067 (7)
O2	0.0174 (8)	0.0138 (8)	0.0276 (8)	0.0042 (6)	0.0098 (6)	0.0035 (6)
O3	0.0152 (8)	0.0248 (10)	0.0348 (10)	0.0051 (7)	0.0099 (7)	0.0120 (8)
O4	0.0198 (8)	0.0152 (9)	0.0338 (9)	0.0032 (7)	0.0051 (7)	−0.0029 (7)
O5	0.0186 (8)	0.0183 (9)	0.0343 (9)	0.0062 (7)	0.0049 (7)	−0.0008 (7)
O6	0.0173 (9)	0.0201 (10)	0.0484 (11)	0.0055 (7)	0.0105 (8)	0.0042 (8)
O7	0.0160 (8)	0.0188 (9)	0.0244 (8)	0.0005 (7)	0.0032 (6)	−0.0025 (7)
O8	0.0174 (8)	0.0170 (9)	0.0302 (9)	0.0039 (7)	0.0105 (7)	−0.0001 (7)
O9	0.0134 (8)	0.0204 (9)	0.0321 (9)	0.0023 (7)	0.0079 (7)	0.0027 (7)
O1w	0.0184 (8)	0.0241 (10)	0.0337 (9)	0.0082 (7)	0.0108 (7)	0.0114 (7)
O2w	0.0316 (10)	0.0196 (9)	0.0203 (8)	0.0097 (8)	0.0058 (7)	0.0005 (7)
O3w	0.0237 (9)	0.0198 (9)	0.0195 (8)	0.0093 (7)	0.0065 (7)	0.0013 (7)
O4w	0.0262 (10)	0.0251 (10)	0.0301 (9)	0.0082 (8)	0.0067 (7)	0.0042 (8)
O5w	0.0298 (10)	0.0359 (12)	0.0292 (10)	0.0122 (9)	0.0072 (8)	−0.0007 (8)
O6w	0.0200 (9)	0.0194 (9)	0.0330 (10)	0.0057 (7)	0.0092 (7)	0.0001 (7)
O7w	0.0404 (11)	0.0183 (9)	0.0272 (9)	0.0055 (8)	0.0109 (8)	0.0021 (7)
O8w	0.0293 (10)	0.0204 (10)	0.0427 (11)	0.0062 (8)	0.0128 (8)	0.0068 (8)
C1	0.0153 (11)	0.0168 (11)	0.0172 (10)	0.0023 (9)	0.0030 (8)	−0.0022 (9)
C2	0.0156 (10)	0.0159 (11)	0.0170 (10)	0.0029 (9)	0.0047 (8)	0.0000 (8)
C3	0.0185 (11)	0.0181 (12)	0.0224 (11)	0.0074 (9)	0.0057 (9)	0.0039 (9)
C4	0.0207 (11)	0.0155 (11)	0.0206 (11)	0.0047 (9)	0.0066 (9)	0.0040 (9)
C5	0.0162 (11)	0.0183 (12)	0.0185 (11)	0.0028 (9)	0.0057 (8)	−0.0004 (9)
C6	0.0180 (11)	0.0180 (12)	0.0222 (11)	0.0066 (9)	0.0055 (9)	0.0046 (9)
C7	0.0190 (11)	0.0155 (11)	0.0182 (10)	0.0028 (9)	0.0046 (8)	0.0029 (9)
C8	0.0181 (11)	0.0172 (12)	0.0189 (10)	0.0026 (9)	0.0044 (8)	−0.0012 (9)
C9	0.0185 (11)	0.0136 (11)	0.0185 (10)	0.0046 (9)	0.0039 (8)	−0.0004 (8)
C10	0.0203 (11)	0.0158 (12)	0.0276 (12)	0.0078 (9)	0.0054 (9)	0.0029 (9)
C11	0.0222 (12)	0.0147 (12)	0.0318 (13)	0.0040 (10)	0.0102 (10)	0.0027 (10)
C12	0.0186 (11)	0.0184 (12)	0.0230 (11)	0.0036 (9)	0.0055 (9)	−0.0021 (9)
C13	0.0215 (12)	0.0185 (12)	0.0247 (12)	0.0082 (10)	0.0023 (9)	0.0034 (9)
C14	0.0216 (12)	0.0156 (12)	0.0245 (11)	0.0032 (10)	0.0064 (9)	0.0022 (9)
C15	0.0141 (10)	0.0166 (11)	0.0176 (10)	0.0052 (9)	0.0031 (8)	0.0036 (8)
C16	0.0147 (10)	0.0166 (11)	0.0142 (10)	0.0043 (9)	0.0021 (8)	0.0016 (8)
C17	0.0163 (11)	0.0163 (11)	0.0189 (10)	0.0055 (9)	0.0051 (8)	0.0024 (9)
C18	0.0188 (11)	0.0148 (11)	0.0221 (11)	0.0030 (9)	0.0053 (9)	0.0008 (9)
C19	0.0121 (10)	0.0209 (12)	0.0200 (11)	0.0036 (9)	0.0040 (8)	0.0051 (9)
C20	0.0175 (11)	0.0190 (12)	0.0217 (11)	0.0070 (9)	0.0064 (9)	0.0001 (9)
C21	0.0163 (11)	0.0161 (11)	0.0189 (10)	0.0026 (9)	0.0028 (8)	−0.0008 (9)

### Geometric parameters (Å, °)

**Table:**

Tb1—O4	2.2791 (17)	O8w—H81	0.836 (10)
Tb1—O7	2.3162 (16)	O8w—H82	0.840 (10)
Tb1—O8^i^	2.3276 (16)	C1—C2	1.480 (3)
Tb1—O2w	2.4042 (17)	C2—C3	1.394 (3)
Tb1—O3w	2.4227 (18)	C2—C7	1.398 (3)
Tb1—O2	2.4276 (16)	C3—C4	1.385 (3)
Tb1—O1	2.4388 (17)	C3—H3A	0.9500
Tb1—O1w	2.4482 (17)	C4—C5	1.386 (3)
O1—C1	1.262 (3)	C4—H4	0.9500
O2—C1	1.280 (3)	C5—C6	1.398 (3)
O3—C5	1.364 (3)	C6—C7	1.383 (3)
O3—H3	0.837 (10)	C6—H6A	0.9500
O4—C8	1.256 (3)	C7—H7	0.9500
O5—C8	1.265 (3)	C8—C9	1.494 (3)
O6—C12	1.370 (3)	C9—C10	1.387 (3)
O6—H6	0.833 (10)	C9—C14	1.395 (4)
O7—C15	1.262 (3)	C10—C11	1.395 (3)
O8—C15	1.262 (3)	C10—H10	0.9500
O8—Tb1^i^	2.3276 (16)	C11—C12	1.375 (4)
O9—C19	1.368 (3)	C11—H11A	0.9500
O9—H9	0.841 (10)	C12—C13	1.389 (4)
O1w—H11	0.839 (10)	C13—C14	1.392 (3)
O1w—H12	0.834 (10)	C13—H13	0.9500
O2w—H21	0.838 (10)	C14—H14	0.9500
O2w—H22	0.832 (10)	C15—C16	1.499 (3)
O3w—H31	0.835 (10)	C16—C21	1.395 (3)
O3w—H32	0.840 (10)	C16—C17	1.397 (3)
O4w—H41	0.837 (10)	C17—C18	1.389 (3)
O4w—H42	0.840 (10)	C17—H17	0.9500
O5w—H51	0.861 (10)	C18—C19	1.394 (3)
O5w—H52	0.852 (10)	C18—H18	0.9500
O6w—H61	0.846 (10)	C19—C20	1.394 (3)
O6w—H62	0.841 (10)	C20—C21	1.394 (3)
O7w—H71	0.842 (10)	C20—H20	0.9500
O7w—H72	0.833 (10)	C21—H21A	0.9500
			
O4—Tb1—O7	147.98 (6)	C4—C3—H3A	119.9
O4—Tb1—O8^i^	86.52 (6)	C2—C3—H3A	119.9
O7—Tb1—O8^i^	97.15 (6)	C3—C4—C5	119.7 (2)
O4—Tb1—O2w	71.93 (6)	C3—C4—H4	120.2
O7—Tb1—O2w	138.23 (6)	C5—C4—H4	120.2
O8^i^—Tb1—O2w	97.25 (6)	O3—C5—C4	122.0 (2)
O4—Tb1—O3w	136.40 (6)	O3—C5—C6	117.2 (2)
O7—Tb1—O3w	74.94 (6)	C4—C5—C6	120.8 (2)
O8^i^—Tb1—O3w	77.43 (6)	C7—C6—C5	119.1 (2)
O2w—Tb1—O3w	70.33 (6)	C7—C6—H6A	120.4
O4—Tb1—O2	76.79 (6)	C5—C6—H6A	120.4
O7—Tb1—O2	83.64 (6)	C6—C7—C2	120.5 (2)
O8^i^—Tb1—O2	147.37 (6)	C6—C7—H7	119.8
O2w—Tb1—O2	103.72 (6)	C2—C7—H7	119.8
O3w—Tb1—O2	133.16 (6)	O4—C8—O5	122.9 (2)
O4—Tb1—O1	108.71 (6)	O4—C8—C9	117.9 (2)
O7—Tb1—O1	78.16 (6)	O5—C8—C9	119.2 (2)
O8^i^—Tb1—O1	158.46 (6)	C10—C9—C14	119.2 (2)
O2w—Tb1—O1	74.23 (6)	C10—C9—C8	120.3 (2)
O3w—Tb1—O1	81.06 (6)	C14—C9—C8	120.4 (2)
O2—Tb1—O1	53.69 (6)	C9—C10—C11	120.4 (2)
O4—Tb1—O1w	78.28 (6)	C9—C10—H10	119.8
O7—Tb1—O1w	73.07 (6)	C11—C10—H10	119.8
O8^i^—Tb1—O1w	70.82 (6)	C12—C11—C10	119.7 (2)
O2w—Tb1—O1w	148.59 (6)	C12—C11—H11A	120.2
O3w—Tb1—O1w	130.93 (6)	C10—C11—H11A	120.2
O2—Tb1—O1w	78.37 (6)	O6—C12—C11	116.8 (2)
O1—Tb1—O1w	126.13 (6)	O6—C12—C13	122.2 (2)
C1—O1—Tb1	93.28 (14)	C11—C12—C13	121.0 (2)
C1—O2—Tb1	93.32 (14)	C14—C13—C12	119.1 (2)
C5—O3—H3	107 (2)	C14—C13—H13	120.5
C8—O4—Tb1	150.98 (16)	C12—C13—H13	120.5
C12—O6—H6	114 (2)	C13—C14—C9	120.6 (2)
C15—O7—Tb1	150.17 (16)	C13—C14—H14	119.7
C15—O8—Tb1^i^	136.88 (15)	C9—C14—H14	119.7
C19—O9—H9	113 (2)	O8—C15—O7	123.1 (2)
Tb1—O1w—H11	125.7 (19)	O8—C15—C16	117.9 (2)
Tb1—O1w—H12	121.8 (19)	O7—C15—C16	119.0 (2)
H11—O1w—H12	109.2 (16)	C21—C16—C17	119.4 (2)
Tb1—O2w—H21	118 (2)	C21—C16—C15	119.8 (2)
Tb1—O2w—H22	110 (2)	C17—C16—C15	120.8 (2)
H21—O2w—H22	110.7 (16)	C18—C17—C16	120.3 (2)
Tb1—O3w—H31	121 (2)	C18—C17—H17	119.8
Tb1—O3w—H32	114.7 (19)	C16—C17—H17	119.8
H31—O3w—H32	109.5 (16)	C17—C18—C19	119.7 (2)
H41—O4w—H42	108.8 (16)	C17—C18—H18	120.1
H51—O5w—H52	105.4 (15)	C19—C18—H18	120.1
H61—O6w—H62	108.3 (16)	O9—C19—C18	122.6 (2)
H71—O7w—H72	108.9 (16)	O9—C19—C20	116.8 (2)
H81—O8w—H82	109.5 (16)	C18—C19—C20	120.6 (2)
O1—C1—O2	119.7 (2)	C21—C20—C19	119.2 (2)
O1—C1—C2	120.5 (2)	C21—C20—H20	120.4
O2—C1—C2	119.7 (2)	C19—C20—H20	120.4
C3—C2—C7	119.6 (2)	C20—C21—C16	120.7 (2)
C3—C2—C1	120.0 (2)	C20—C21—H21A	119.7
C7—C2—C1	120.2 (2)	C16—C21—H21A	119.7
C4—C3—C2	120.2 (2)		
			
O4—Tb1—O1—C1	−56.96 (14)	C3—C4—C5—C6	3.5 (3)
O7—Tb1—O1—C1	90.56 (14)	O3—C5—C6—C7	176.6 (2)
O8^i^—Tb1—O1—C1	169.90 (15)	C4—C5—C6—C7	−3.4 (3)
O2w—Tb1—O1—C1	−121.11 (14)	C5—C6—C7—C2	0.5 (3)
O3w—Tb1—O1—C1	166.92 (14)	C3—C2—C7—C6	2.3 (3)
O2—Tb1—O1—C1	−0.22 (12)	C1—C2—C7—C6	−171.8 (2)
O1w—Tb1—O1—C1	31.99 (16)	Tb1—O4—C8—O5	−108.1 (3)
O4—Tb1—O2—C1	125.77 (14)	Tb1—O4—C8—C9	72.0 (4)
O7—Tb1—O2—C1	−79.74 (13)	O4—C8—C9—C10	−156.2 (2)
O8^i^—Tb1—O2—C1	−173.07 (12)	O5—C8—C9—C10	23.8 (3)
O2w—Tb1—O2—C1	58.44 (14)	O4—C8—C9—C14	21.5 (3)
O3w—Tb1—O2—C1	−17.32 (16)	O5—C8—C9—C14	−158.5 (2)
O1—Tb1—O2—C1	0.22 (12)	C14—C9—C10—C11	−1.5 (4)
O1w—Tb1—O2—C1	−153.71 (14)	C8—C9—C10—C11	176.2 (2)
O7—Tb1—O4—C8	66.9 (4)	C9—C10—C11—C12	1.9 (4)
O8^i^—Tb1—O4—C8	−31.0 (3)	C10—C11—C12—O6	179.8 (2)
O2w—Tb1—O4—C8	−129.8 (3)	C10—C11—C12—C13	−0.1 (4)
O3w—Tb1—O4—C8	−98.7 (3)	O6—C12—C13—C14	178.0 (2)
O2—Tb1—O4—C8	120.8 (3)	C11—C12—C13—C14	−2.1 (4)
O1—Tb1—O4—C8	164.6 (3)	C12—C13—C14—C9	2.4 (4)
O1w—Tb1—O4—C8	40.1 (3)	C10—C9—C14—C13	−0.7 (4)
O4—Tb1—O7—C15	169.6 (3)	C8—C9—C14—C13	−178.4 (2)
O8^i^—Tb1—O7—C15	−95.6 (3)	Tb1^i^—O8—C15—O7	14.8 (4)
O2w—Tb1—O7—C15	13.8 (3)	Tb1^i^—O8—C15—C16	−163.99 (16)
O3w—Tb1—O7—C15	−20.7 (3)	Tb1—O7—C15—O8	81.1 (4)
O2—Tb1—O7—C15	117.3 (3)	Tb1—O7—C15—C16	−100.2 (3)
O1—Tb1—O7—C15	63.1 (3)	O8—C15—C16—C21	167.8 (2)
O1w—Tb1—O7—C15	−163.0 (3)	O7—C15—C16—C21	−11.1 (3)
Tb1—O1—C1—O2	0.4 (2)	O8—C15—C16—C17	−10.6 (3)
Tb1—O1—C1—C2	−176.78 (18)	O7—C15—C16—C17	170.5 (2)
Tb1—O2—C1—O1	−0.4 (2)	C21—C16—C17—C18	−0.9 (3)
Tb1—O2—C1—C2	176.80 (18)	C15—C16—C17—C18	177.6 (2)
O1—C1—C2—C3	−7.7 (3)	C16—C17—C18—C19	0.0 (3)
O2—C1—C2—C3	175.1 (2)	C17—C18—C19—O9	−179.5 (2)
O1—C1—C2—C7	166.3 (2)	C17—C18—C19—C20	1.2 (4)
O2—C1—C2—C7	−10.8 (3)	O9—C19—C20—C21	179.2 (2)
C7—C2—C3—C4	−2.3 (3)	C18—C19—C20—C21	−1.5 (4)
C1—C2—C3—C4	171.8 (2)	C19—C20—C21—C16	0.5 (3)
C2—C3—C4—C5	−0.6 (4)	C17—C16—C21—C20	0.6 (3)
C3—C4—C5—O3	−176.5 (2)	C15—C16—C21—C20	−177.8 (2)

Symmetry codes: (i) −*x*+1, −*y*+1, −*z*+1.

### Hydrogen-bond geometry (Å, °)

**Table:**

*D*—H···*A*	*D*—H	H···*A*	*D*···*A*	*D*—H···*A*
O3—H3···O7w^ii^	0.84 (1)	1.82 (2)	2.612 (3)	158 (3)
O6—H6···O6w^iii^	0.83 (1)	1.87 (1)	2.678 (3)	165 (4)
O9—H9···O4w^iv^	0.84 (1)	1.98 (2)	2.763 (3)	156 (3)
O1w—H11···O4w	0.84 (1)	2.04 (1)	2.870 (3)	171 (3)
O1w—H12···O9^v^	0.83 (1)	1.96 (1)	2.766 (3)	164 (3)
O2w—H22···O5w	0.83 (1)	1.86 (1)	2.678 (3)	167 (3)
O2w—H21···O5w^vi^	0.84 (1)	2.20 (2)	2.952 (3)	150 (3)
O3w—H31···O3^iii^	0.84 (1)	1.95 (1)	2.777 (3)	171 (3)
O3w—H32···O7^i^	0.84 (1)	2.25 (2)	2.916 (2)	137 (3)
O4w—H41···O8w^vii^	0.84 (1)	1.94 (1)	2.753 (3)	163 (3)
O4w—H42···O5^viii^	0.84 (1)	2.10 (1)	2.927 (3)	169 (3)
O5w—H52···O7w	0.85 (1)	2.01 (2)	2.811 (3)	157 (3)
O5w—H51···O8w^ix^	0.86 (1)	1.96 (1)	2.791 (3)	163 (3)
O6w—H61···O2	0.85 (1)	1.90 (1)	2.743 (2)	172 (3)
O6w—H62···O5	0.84 (1)	1.95 (1)	2.785 (2)	171 (3)
O7w—H71···O6w	0.84 (1)	1.90 (1)	2.732 (3)	172 (3)
O7w—H72···O5^x^	0.83 (1)	1.89 (1)	2.725 (3)	175 (3)
O8w—H81···O1^xi^	0.84 (1)	2.10 (2)	2.818 (3)	144 (3)
O8w—H82···O6	0.84 (1)	1.90 (1)	2.713 (3)	164 (3)

Symmetry codes: (ii) −*x*+2, −*y*+1, −*z*; (iii) *x*−1, *y*, *z*; (iv) *x*+1, *y*+1, *z*; (v) −*x*+2, −*y*+1, −*z*+1; (vi) −*x*+1, −*y*+1, −*z*; (i) −*x*+1, −*y*+1, −*z*+1; (vii) −*x*, −*y*, −*z*+1; (viii) −*x*+1, −*y*, −*z*+1; (ix) −*x*, −*y*, −*z*; (x) −*x*+1, −*y*, −*z*; (xi) *x*−1, *y*−1, *z*.
